# Hemoadsorption: a new tool in neurotoxic poisoning

**DOI:** 10.3389/fneph.2025.1647025

**Published:** 2025-10-23

**Authors:** J. Hernandez-Vaquero, A. Repilado-Alvarez, J. C. de la Flor, T. Mata Forte

**Affiliations:** ^1^ Nephrology Department, Hospital Central de la Defensa “Gómez Ulla”, CSVE, Madrid, Spain; ^2^ Spanish Defence Institute of Toxicology, Madrid, Spain; ^3^ Infectious Diseases Department and CBRN Defence Unit, Hospital Central de la Defensa “Gómez Ulla”, CSVE, Madrid, Spain

**Keywords:** hemoperfusion, chemical warfare agents, pesticides/poisoning, renal replacement therapy, organophosphate poisoning

## Abstract

Although the use of neurotoxic agents as weapons of war (CWAs) or in terrorist attacks is relatively uncommon, it has been documented on several occasions in recent history, including the Syrian civil war, the Tokyo subway attack, and the Salisbury incident. The toxidrome associated with these agents is well described; however, treatment remains largely supportive, as effective antidotes are not currently available. Conventional renal replacement therapies (RRT), such as hemodialysis or continuous modalities, are not recommended for managing neurotoxic agent poisoning due to their toxicodynamic properties. In contrast, hemoadsorption (HA), especially when combined with CRRT, has shown promise in organophosphate (OP) pesticide poisonings. Given the chemical similarities between neurotoxic CWAs and OP, HA may represent a rational therapeutic option in selected cases. Notably, substantial differences exist among these agents in terms of onset of action, routes of exposure, and pharmacodynamics, which critically affect both treatment effectiveness and the availability of a therapeutic window. While the management of such exposures has traditionally fallen under military medical services, documented use in terrorist contexts underscores the importance of civilian healthcare professionals being familiar with current treatment options. This article reviews the pathophysiological mechanisms and key chemical properties of neurotoxic agents and evaluates the potential role of HA as an adjunctive therapy in the management of patients exposed to these CWAs.

## Introduction

Although chemical substances have been known as weapons of war since ancient times, they were not used in a structured manner until World War I. The use of chlorine gas in the Second Battle of Ypres and mustard gas (yperite) in the Third Battle highlighted the urgent need for protective respiratory equipment and isolation suits for soldiers exposed to chemical warfare agents (CWAs). Neurotoxic agents (NAs), developed after World War II, marked a qualitative leap in chemical warfare due to their high lethality (1, 2).

Unfortunately, the use of CWAs during the Iran-Iraq war in the 1980s, more recently in the Syrian civil war, the sarin attack on the Tokyo subway, and the attempted assassination of Sergei Skripal, among others, have underscored the necessity for both military and civil defense units to implement protocols and readiness strategies for chemical attacks (3).

While logistical recommendations for protection and decontamination against CWAs are well established (2), the medical management remains insufficiently understood, particularly concerning the “*in vivo*” activity and efficacy of certain treatments for CWAs intoxication. In most cases, supportive care is the only option due to the absence of effective antidotes (4).

Conventional renal replacement therapies (RRT) such as hemodialysis may be appropriate for some poisonings but are not generally recommended for NA or other CWAs intoxications (5). Some conditions related to CWAs intoxications, such as liver failure, implication of protein-bound and non–water-soluble compounds, among others, may justify hemoadsorption (HA) as a possible therapy (6). HA is an emerging extracorporeal therapy used in cases of organophosphate (OP) pesticide poisoning (7), and may represent a supportive therapeutic strategy for patients affected by CWAs.

This article analyzes the pathophysiology and main chemical properties of neurotoxic agents that may justify the potential indications for hemoadsorptive therapy in these intoxications.

## Methods

Due to the scarcity of published literature, a systematic review was not conducted. Instead, a narrative review approach was adopted to synthesize relevant evidence and theoretical frameworks on the use of HA in OP and NA poisoning.

A bibliographic search was conducted in PubMed and the Cochrane Library on March 1, 2025, with no date or country restrictions. However, only full-text articles published in English were included. The following combination of MeSH terms and keywords was used in PubMed:

“Organophosphate Poisoning” OR “Organophosphates” OR “Organophosphate Toxicity” OR “Organophosphate Intoxication” OR “Pesticide Poisoning” AND “Hemoadsorption” OR “Hemoperfusion” OR “Extracorporeal Adsorption” OR “Blood Purification” OR “Extracorporeal Detoxification”

This search yielded 47 results in PubMed. After title and abstract screening, 22 articles were excluded for the following reasons:

- 10 were written in languages other than English
- 1 was not accessible in full text
- 11 were irrelevant to the specific focus of this review or presented redundant content

A total of 25 PubMed articles were included in the final analysis.

In parallel, a search was performed in the Cochrane Library using the following query: “organophosphate” OR “organophosphorus” OR “pesticide poisoning” AND “hemoadsorption” OR “hemoperfusion” OR “blood purification” OR “extracorporeal detoxification”. This search identified 7 relevant clinical trials and 1 protocol entitled “Extracorporeal blood purification for organophosphorus pesticide poisoning.” No completed Cochrane systematic review was found at the time of writing. These additional records were reviewed and considered where applicable to the clinical scope of the article.

**Figure 1:**
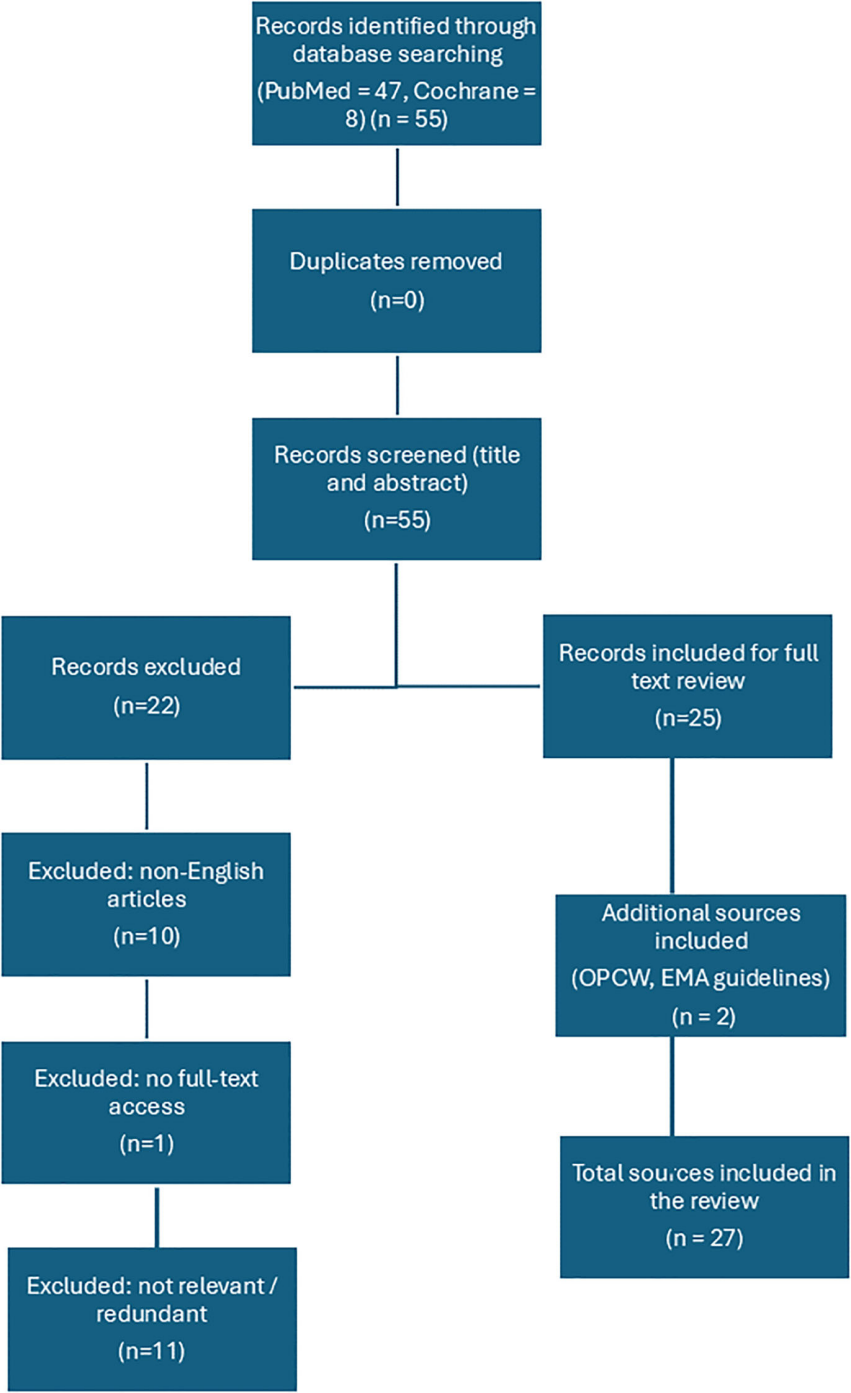
PRISM-style flow diagram of the literature selection process.

The selection process is summarized in a PRISMA-style flow diagram (**Figure 1**) and a summary table. (**Table 1**).

**Table 1:** Summary of included studies on hemoadsorption and extracorporeal therapies in organophosphate intoxication.

Author(s)	Year	Study type	Intervention/focus	Main findings	Relevance to review
Cheng et al.	2025	Clinical trial	HA in OP intox.	Better in HA group	High
Yao et al.	2025	Review	PlasmaE & HA in OP intox.	Better in HA+PE group	Medium
Zhang et al. (8)	2022	Meta-analysis	HA in OP intox.	Better in HA group	High
Sukumar et al.	2019	Review	HA in paraquat intox.	Better in HA group	Low
Guo et al.	2018	Clinical trial	HA in OP intox.	Useful technique	Low
Li et al. (9)	2017	Clinical trial	HA in OP intox.	Better in HA group	High
Ozaki et al.	2017	Case report	HA in OP intox.	Useful technique	Low
Dong et al.	2017	Clinical trial	HA in OP intox.	Better in HA group	High
Liang & Zhang	2015	Clinical trial	HA & PCH in OP intox	Better in HA group	low
Bo	2014	Clinical trial	HA frequency in OP intox.	Repeated HA better than single HA	High
Liu & Ding	2015	Clinical trial	RRT &OP intox.	Better on-line therapy	medium
Nikolaev & Samsonov	2014	Review	HA in OP intox.	Useful technique	Low
Hu et al.	2014	Clinical trial	RRT & HA in OP intox.	HA & SLED better	High
Knežević et al.	2012	Case report	HD & OP intox.	Better in HA group	Low
Kang et al.	2009	Retrospective study	Prognostic risk factors in OP intox.	Not relevant for this review	Low
Schrickel et al.	2009	Case Report	HA in OP intox.	Logistic strategies	Low
Peter et al.	2007	Review	HA in OP intox.	Useful technique	Low
Roberts et al.	2007	Letter to editor	HA in OP intox.	Changes in concentration during HA	Low
Altintop et al.	2005	Clinical trial	HA in OP intox.	Better in HA group	High
Peng et al.	2004	Clinical trial	HA & DDVP	Better in HA group	High
Sakata et al.	1999	Case report	OP intox. & metabolites	Metabolites can vary from animals to humans	High
Martinez-Chuecos et al.	1992	Clinical trial	HA in OP intox.	No differences between groups	High
Köppel et al.	1991	Case Report	HA in Bromophos intox.	Toxic Clearance	High
Kojima et al.	1990	Case Report	HA in Formothion intox.	Toxic Clearance	High
Burgess & Audette	1990	*In vitro* study	Charcoal absorption for malation	Clearance	High

## Results

After removing duplicates and excluding articles unrelated to the topic, a group of nephrologists and specialists in chemical warfare reviewed and identified 25 relevant records. In addition, treatment guidelines published by the Organization for the Prohibition of Chemical Weapons (OPCW) and the European Medicines Agency (10) were included due to their practical relevance.

## Discussion

### Pathophysiology of NAs poisoning

NAs are compounds chemically similar to civilian-use pesticides but modified to increase lethality (10). Their toxicity relies on covalent binding to acetylcholinesterase (AChE), inhibiting its function and causing a cholinergic crisis. However, clinical outcomes vary depending on the structural groups attached to the central phosphorus atom (4, 11).

Three groups of NA agents are described: Group G (sarin, soman, tabun), Group V (VX and related), and Group A (Novichok and related) (10). While “*in vitro*” toxicity can be estimated, the actual toxicological impact of an NA compound also depends on factors such as route of exposure, chemical stability, lipid affinity and thus tissue distribution (4). These parameters, while theoretical in nature, have critical implications for toxicity, symptom duration, and post-exposure management. For instance, inhalational exposure to volatile agents like sarin differs greatly from the transdermal exposure typically seen with less volatile agents like VX from a pharmacodynamic standpoint, which has significant clinical implications (12).

These toxicodynamic differences in agent behavior—along with lipid affinity, chemical stability, and both volume and kinetics of distribution—are key determinants of their clinical effects. Animal studies have shown that inhaled sarin leads to rapid distribution and elimination, whereas percutaneous VX exposure results in a gradual increase in blood concentration with a plateau of at least five hours (13).

Post-inhibition behavior of NAs also affects treatment efficacy, as rapid “aging” of AChE limits the effectiveness of oxime-based reactivators (14). These toxicodynamic differences influence not only clinical severity but also duration of antidotal and supportive therapy, as well as the potential therapeutic window (4).

### Conventional treatment of OP and NA poisoning

Standard treatment for OP poisoning includes supportive care and administration of atropine, oximes, and benzodiazepines. Atropine and benzodiazepines offer only symptomatic relief. Atropine blocks muscarinic parasympathetic symptoms and often requires high initial doses followed by continuous infusion until toxic effects are reversed (10). Despite its availability, large-scale events could exhaust existing medical supplies.

Oximes are intended to reactivate AChE, but their efficacy, especially pralidoxime and obidoxime, against certain NAs like Novichok is questioned in the literature (15). Both OPs and NAs can cross the blood-brain barrier, leading to persistent seizures through cholinergic receptor overstimulation, GABA suppression, and glutamatergic hyperactivation, potentially resulting in long-term neurological damage. Benzodiazepines, particularly fast-acting agents such as midazolam, may offer neuroprotection in this context (16).

### HA and toxin removal

HA is an extracorporeal technique that enables the removal of harmful circulating molecules through weak chemical interactions, such as hydrophobic, ionic, and Van der Waals forces, with an adsorbent material. It is an alternative to dialysis in situations where this treatment is not effective (for example, intoxication with protein-bound toxins or drugs or poisoning with non–water-soluble toxins).

Current HA cartridges use cross-linked divinylbenzene polymers, structured into beads and often coated with polysulfone to enhance biocompatibility. These structures provide adsorption surfaces exceeding 1000 m²/g, with cartridges containing 200–300g of sorbent (6).

Patient blood is exposed to the sorbent in a dialysis-like circuit, which can be used adjunctively with standard RRT. While clinical experience is limited, HA is increasingly used in sepsis and cardiac surgery to reduce circulating cytokines and endotoxins. It has also shown utility in removing excess levels of certain drugs such as apixaban, carbamazepine, or myoglobin. However, the lack of randomized clinical trials limits broader adoption (17).

OP poisoning is considered an indication for HA. A 2022 meta-analysis by Zhang et al. that included a total of 11 randomized controlled trials with 811 patients, compared outcomes in patients treated with standard RRT alone versus combined RRT and HA. Results favored HA in reducing mortality, hospital stay duration, mechanical ventilation needs, atropine dosage, and AChE recovery time (8).

Theoretically, lipophilic toxins with medium-to-high molecular weight and significant protein binding may be suitable for HA removal, particularly when resin-based sorbents are used. In contrast, toxins with high volume of distribution and slow intercompartmental kinetics are less amenable to RRT and HA (18).

While the octanol-water partition coefficient (LogP) helps estimate lipophilicity, it does not reliably predict HA efficacy alone. Molecular weight (MW) seems less influential, although HA performs better than traditional RRT in clearing medium- to high-MW molecules (19).

HA is only capable of removing the NA from the vascular space. However, when combined with oximes and RRT, it can reduce the acute cholinergic phase and significantly contribute to the restoration of fluid, electrolyte, and acid-base homeostasis associated with the toxidrome (8).

For NA poisoning, the therapeutic relevance of HA may depend on agent-specific toxicodynamics, particularly regarding AChE aging rates and the duration the NT remains in the bloodstream. As previously mentioned, the expected kinetics in poisoning with agents such as VX suggest a stable neurotoxin concentration in blood for several hours (13), thereby making its removal through extracorporeal purification techniques a feasible intervention. Moreover, the mechanism of action of NA involves the phosphorylation of the serine hydroxyl group in the active site of AChE by the organophosphate compound. Initially, this phosphorylated enzyme can be reactivated by nucleophilic agents such as oximes.

However, over time, a secondary chemical process known as “aging” may occur. Aging refers to the dealkylation of one of the side chains on the phosphorus atom of the OP moiety covalently bound to AChE. This reaction results in the formation of a more stable phosphate-enzyme complex, refractory to reactivation by oximes or other nucleophiles.

The rate at which aging occurs varies significantly depending on the chemical structure of the OP compound. For instance, soman causes enzyme aging within minutes, whereas VX may take up to 37 hours (14).

This kinetic variability may have important implications for the use of extracorporeal therapies such as HA. HA aims to remove circulating toxic agents from the bloodstream before they exert irreversibly damage. If aging occurs rapidly, a significant proportion of AChE may become irreversibly inhibited before the OP is cleared from the circulation, limiting the clinical utility of HA.

In cases where aging is delayed, HA could theoretically reduce the plasma concentration of the parent OP compound, thereby decreasing the amount available to inhibit AChE and potentially preserving enzymatic activity (20).

Beyond these considerations, some agents, especially Group V compounds, require hepatic metabolism via cytochrome P450 enzymes to generate toxic metabolites, unlike sarin, which acts directly (21), so part of the toxin or its circulating metabolites can be removed from the circulation with HA, decreasing the absorbed dose and reducing its systemic effects.

### Evaluation of NT agents and HA compatibility

#### Group G agents

Sarin, soman and tabun exhibits moderate lipophilicity, allowing rapid diffusion across membranes and the blood-brain barrier.

Sarin is a low-molecular weight (MW) (140 Da), moderately lipophilic molecule (LogP 0.3), diffusible across membranes, but water-soluble, implying low protein binding. Its high volatility favors inhalational absorption, leading to brief blood presence and rapid CNS action, with limited feasibility for extracorporeal clearance. It is rapidly hydrolyzed by plasma and hepatic esterases into non-toxic metabolites excreted renally (22).

Soman, slightly heavier and more lipophilic (LogP 1.78), also shows rapid AChE aging, making HA unlikely to offer clinically meaningful removal (23, 24).

Therefore, since NAs belonging to this group are not expected to remain in the bloodstream long enough to allow the implementation of extracorporeal purification therapy in the affected patient, and given the rapid aging process of AChE, the role of HA is likely to be very limited.

#### Group V agents

VX and related molecules have also low MW (~268 Da), high lipophilicity (LogP >2), and large volume of distribution. After binding to AChE, the “aging” process is slower than in Group G compounds and may take up to 37 hours, allowing a therapeutic window. There is also accumulation of the NA in the body’s adipose tissue, which may explain the presence of stable blood concentrations of the agent over several hours (13). These characteristics suggest that patients poisoned with VX or related may benefit from HA as an adjunct to standard treatment (14, 25, 26).

#### Group A

Novichok agents, banned by OPCW after the 2018 Salisbury attack (15), share pharmacodynamic characteristics with Group V but are 5 to 8 times more toxic. They may be absorbed via inhalation and transdermally. Oxime therapies have shown limited efficacy (3, 26). Novichok agents have a low-medium MW and moderate-to-high lipophilicity but may exhibit delayed kinetics over several hours when absorption occurs through the skin. Despite the limited understanding of the toxidrome associated with substances in this group, their chemical characteristics and laboratory-observed kinetics indicate that removal via HA could be a viable therapeutic option (27) (see **Table 2**).

**Table 2:** Physicochemical properties of NT agents and theoretical compatibility with HA.

Group	Compound	MW (Da)	LogP (lipophilicity)	Dominant Absorption Route	AChE Aging Rate	Volume of Distribution	Theoretical HA Compatibility	Brief Justification
G	Sarin	~140	0.3 (low-moderate)	Inhalation (high volatility)	Rapid (minutes)	Low	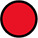 Very Low	Short half-life in blood; early central effect.
G	Soman	~180	1.78 (high)	Inhalation or dermal	Very rapid (minutes)	Moderate	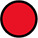 Very Low	Rapid AChE aging, limited therapeutic window.
G	Tabun	~162	~1.2 (estimated)	Similar to soman	Rapid	Moderate	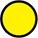 Low	Slightly more stable than soman, but still limited efficacy.
V	VX	~267	>2 (high)	Transdermal (low volatility)	Slow (up to 37 h)	High	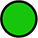 Moderate - High	Lipophilic, tissue accumulation; possible HA window.
A	Novichok	~270–290	Moderate-high (2–3)	Inhalation and transdermal	Variable, slow in some cases	High	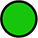 Moderate	Similar to VX, higher toxicity; theoretical HA possible.

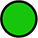
 = High potential for HA 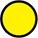 = Moderate potential for HA 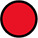 = Low or negligible potential for HA.

### Intermediate syndrome and NAs

IS is a delayed-onset neurological syndrome involving respiratory paralysis and cranial nerve dysfunction (III, IV, X), observed in a subset of OP poisonings (28). It is currently assumed that CWAs do not cause this syndrome (10), which may be related more to the limited number of analyzed cases involving NTs and their high associated lethality than to chemical differences. However, some studies show that HA combined with conventional treatment reduces IS incidence in OP poisoning (9). Given the limited clinical experience in the management of CWA poisonings, it remains unclear whether patients receiving intensive care for NT intoxication are at increased risk of developing IS. It is plausible that, due to improved survival associated with intensive supportive therapy, these patients may live long enough to manifest delayed complications such as IS. Nevertheless, drawing on clinical experience with OP poisoning (9), it is reasonable to hypothesize that the use of HA in combination with RRT may reduce the incidence of IS in NT intoxications. Therefore, early consideration of HA as an adjunctive therapy may be warranted in the clinical management of these patients.

## Limitations

Only publicly available data sources were included, as production, possession, and use of these agents are internationally banned (15). Some relevant studies may remain classified or unpublished.

No randomized trials or large case series exist regarding CWA effects on humans. Some physicochemical data were derived from computational models, and clinical data from limited case reports. While HA is commonly used in clinical settings, most trials focus on sepsis or cardiac surgery, limiting evidence for intoxication scenarios.

## Conclusions

Although intoxication with NAs is rare, it has been documented on multiple occasions in recent history. Owing to its high lethality, effective medical response remains a significant challenge. Conventional therapy with atropine, oximes and benzodiazepines offers symptomatic relief but limited efficacy. Given its demonstrated utility in OP poisoning, HA using polymer resin cartridges, may enhance the elimination of NAs and NA–antidote complexes, potentially reducing the incidence of IS incidence among survivors of the acute cholinergic phase. Currently, RRT and HA is a technique available in nearly all hospital centers in our setting. Moreover, with the use of home hemodialysis monitors, it is now possible to perform renal purification therapies or SLED-like therapies even in out-of-hospital environments (see **Box 1**). Therefore, any technique that can safely improve the prognosis, reduce the need for intensive care, and decrease antidote requirements in patients affected by NA exposure is considered valuable. Nevertheless, the efficacy of HA in cases of CWAs exposure remains unproven, and further *in vitro* studies are necessary to validate its therapeutic potential.

Box 1 **Clinical recommendation box**

• Hemoadsorption (HA) has shown clinical benefit in organophosphate (OP) poisoning by reducing antidote requirements and ICU length of stay.

• Since nerve agents (NAs) are OP compounds, their removal through extracorporeal HA is theoretically plausible.

• The efficacy of HA depends on the physicochemical properties of the agent. Compounds with high lipophilicity and slow acetylcholinesterase (AChE) aging kinetics (e.g., VX, Novichok) are more suitable for removal.

• HA should be initiated as early as possible, ideally in combination with renal replacement therapy (RRT), and always alongside standard antidotal treatment.

• Consider HA particularly in suspected exposures to NAs from groups A and V (e.g., Novichok, VX).

• The use of HA may also be feasible in field hospitals or out-of-hospital settings using home hemodialysis devices, provided appropriate training and equipment are available.
